# Bone Marrow Stromal Antigen 2 (BST-2) DNA Is Demethylated in Breast Tumors and Breast Cancer Cells

**DOI:** 10.1371/journal.pone.0123931

**Published:** 2015-04-10

**Authors:** Wadie D. Mahauad-Fernandez, Nicholas C. Borcherding, Weizhou Zhang, Chioma M. Okeoma

**Affiliations:** 1 Department of Microbiology, Carver College of Medicine, University of Iowa, Iowa City, Iowa, United States of America; 2 Interdisciplinary Graduate program in Molecular and Cellular Biology (MCB), University of Iowa, Iowa City, Iowa, United States of America; 3 Department of Pathology, Carver College of Medicine, University of Iowa, Iowa City, Iowa, United States of America; 4 Medical Scientist Training Program, Carver College of Medicine, University of Iowa, Iowa City, Iowa, United States of America; University of North Carolina School of Medicine, UNITED STATES

## Abstract

**Background:**

Bone marrow stromal antigen 2 (BST-2) is a known anti-viral gene that has been recently identified to be overexpressed in many cancers, including breast cancer. BST-2 is critical for the invasiveness of breast cancer cells and the formation of metastasis *in vivo*. Although the regulation of BST-2 in immune cells is unraveling, it is unknown how BST-2 expression is regulated in breast cancer. We hypothesized that meta-analyses of BST-2 gene expression and BST-2 DNA methylation profiles would illuminate mechanisms regulating elevated BST-2 expression in breast tumor tissues and cells.

**Materials and Methods:**

We performed comprehensive meta-analyses of BST-2 gene expression and BST-2 DNA methylation in The Cancer Genome Atlas (TCGA) and various Gene Expression Omnibus (GEO) datasets. BST-2 expression levels and BST-2 DNA methylation status at specific CpG sites on the BST-2 gene were compared for various breast tumor molecular subtypes and breast cancer cell lines.

**Results:**

We show that BST-2 gene expression is inversely associated with the methylation status at specific CpG sites in primary breast cancer specimens and breast cancer cell lines. BST-2 demethylation is significantly more prevalent in primary tumors and cancer cells than in normal breast tissues or normal mammary epithelial cells. Demethylation of the BST-2 gene significantly correlates with its mRNA expression. These studies provide the initial evidence that significant differences exist in BST-2 DNA methylation patterns between breast tumors and normal breast tissues, and that BST-2 expression patterns in tumors and cancer cells correlate with hypomethylated BST-2 DNA.

**Conclusion:**

Our study suggests that the DNA methylation pattern and expression of BST-2 may play a role in disease pathogenesis and could serve as a biomarker for the diagnosis of breast cancer.

## Introduction

Breast cancer is the second largest cause of cancer-related deaths in women according to the National Cancer Institute (NCI) and is the second most common cancer diagnosed in women. Treatment for breast cancer is dependent on its subtype classification [1]. The most severe forms of breast cancer which respond poorly to hormonal or targeted therapies include luminal B and basal breast cancers [2,3]. The inability to develop new treatments is partially due to a limited understanding of all the drivers of these malignancies which give transformed cells a selective advantage over normal cells to grow and thrive in unfavorable environments.

One of the drivers of breast malignancy is BST-2 [4], also known as Tetherin, CD317, or HM1.24. BST-2 is an IFN-inducible type II transmembrane protein expressed mainly at the surface of cells [5,6]. BST-2 contains an N-terminus cytoplasmic tail followed by a transmembrane domain, an extracellular coiled-coiled domain and a C-terminus glycophosphatidylinositol (GPI) anchor embedded in lipid rafts along the cell membrane [5,7]. BST-2 was discovered as a marker of differentiated B cells [8] and was later rediscovered as a potent antiviral restriction factor with the ability to tether enveloped viruses to the cell membrane of infected cells via its GPI anchor [9–12], as well as to potently inhibit virus replication in cultured cells and *in vivo* [11,13,14]. BST-2 is thought to mediate host immune response by activating NF-кB through interaction with transforming growth factor beta-activated kinase 1(TAK1) and TNF receptor associated factors (TRAFs) 2 and 6 [14–16]. In addition, BST-2 induces antibody-dependent cell cytotoxicity (ADCC) against the envelope protein of HIV [17–19].

Recent studies have demonstrated that the mRNA and protein expression of BST-2 are elevated in various cancers including: head and neck cancer, oral cavity cancer, glioblastoma, lung cancer, endometrial cancer, lymphomas, and breast cancers [20–26]. There is direct evidence for a role for BST-2 in two cancers. BST-2 antibody-mediated ADCC has been shown to be potent in myeloma treatment [27,28] and in breast cancer, BST-2 plays a direct role in driving breast malignancy [4]. In vivo, elevated BST-2 expression is associated with primary tumor growth, metastasis, and poor prognosis [4]. Upon BST-2 knockdown, breast cancer cells lose their capacity to grow and thrive *in vivo* [4]. The molecular mechanisms involved in BST-2-mediated breast cancer malignancy includes; BST-2-meditated enhancement in cancer cell i) adhesion to the tumor microenvironment, ii) migration through the basement membrane, iii) invasion through extracellular matrix lattice, and anchorage independent growth [4,29]. In contrast to breast cancer, knock down of BST-2 in glioblastoma had no effect on tumor growth in mice [22].

Despite the functions of BST-2 in breast oncogenesis, little is known about the regulation of BST-2 expression in cancer cells. Sayeed *et al*., (2013) reported that BST-2 expression in tumor tissues and primary breast cancer cell lines is negatively regulated by transforming growth factor beta (TGF-β) [30]. However, there is no evidence for genetic or epigenetic modifications that regulate BST-2 expression in breast cancer tissue/cells.

The process of carcinogenesis is characterized by genetic and epigenetic modifications. Epigenetic alterations and regulation of gene functions is increasingly being recognized as critical in carcinogenesis [31]. These alterations may involve histone modifications and changes in DNA methylation status of cytosine bases (C) in the context of CpG dinucleotides.

The result of alterations in DNA methylation status is changes in gene expression patterns that may perturb normal cell physiology and function. There is an inverse correlation between gene expression and DNA methylation status [32,33]. As such, hypermethylation of DNA silences gene expression [32] whereas hypomethylation or demethylation of DNA enables gene expression [34]. Both hypermethylation and hypomethylation play important but distinct roles in the initiation, progression, and metastasis of various cancers [35,36]. Here, we aimed to determine the source of BST-2 overexpression in breast tumors through *in silico* and *in vitro* analyses. We report that BST-2 expression in breast tumors and cancer cells is epigenetically regulated by hypomethylation or demethylation of specific CpG sites along the BST-2 gene.

## Methods

### Cell lines

Normal human mammary epithelial cell line HMLE, luminal A breast cancer cell lines MCF-7 and T47D, luminal B cell line BT-474, HER2+ cell line SK-BR-3, triple negative MDA-MB-231 cell line, and basal breast cancer cell line SUM-159 are from ATCC and were maintained according to ATCC instructions.

### Gene expression and methylation analysis

**Fig 1:**
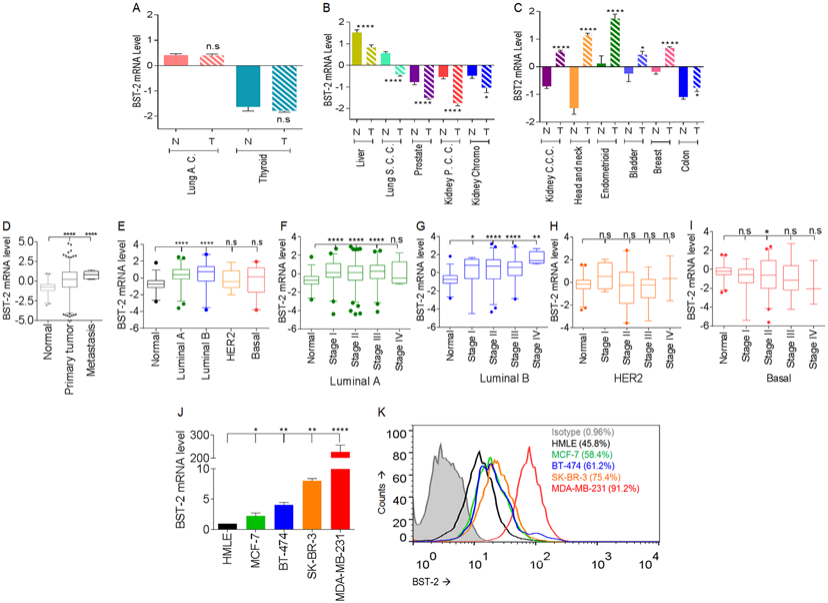
Various cancer types including breast cancer have different levels of BST-2 expression. (A) to (C) BST-2 transcript levels in paired normal (N) and tumor (T) tissues of patients bearing different cancer types. Data analyzed are from TCGA. Lung adenocarcinoma = Lung A. C., Lung Squamous Cell Carcinoma = Lung S. C. C., Kidney Papillary Cell Carcinoma = Kidney P. C. C., Kidney Clear Cell Carcinoma = Kidney C. C. C. (D) BST-2 mRNA levels of normal, primary tumor, and metastatic tumor of invasive breast cancer bearing patients from TCGA repository. (E) BST-2 mRNA levels in different breast cancer subtypes. BST-2 levels in all tumor subtypes are compared to the BST-2 level in normal tissues. Data does not include metastatic tumors. (F) to (I) Box plots of BST-2 transcript levels in stages I, II, III, and IV of (F) luminal A, (G) luminal B, (H) HER2-enriched, and (I) basal breast cancers. Data does not include metastatic tumors. (J) BST-2 mRNA levels in different breast cancer cell lines. BST-2 levels in all cancer cell lines are compared to BST-2 levels in normal HMLE cells and normalized to GAPDH. (K) BST-2 surface expression from HMLE, MCF-7, BT-474, SK-BR-3 and MDA-MB-231 cells as determined by flow cytometry. Numbers in parenthesis correspond to BST-2 expression presented as a percentage. Significance was taken at P<0.05 (*), P<0.01 (**) and P<0.001 (****). Error bars correspond to standard error of the mean (SEM) and n.s = not significant.

The UCSC Cancer Genome Browser (https://genome-cancer.ucsc.edu) [37] was used to assess BST-2 expression for the PAN-CAN-normalized samples [38,39] for the indicated cancer types and their corresponding normal tissues. Separately, expression and methylation values from the individual BRCA cohort of the TCGA were used. Expression versus methylation analyses were performed with mean-centralized level 3 Illumina HiSeq 2000 RNAseq expression data and Infinium HumanMethylation450 beta-values. Methylation beta-values are reported as either an average of all probes or by the specific probe for BST-2. Probes on the BST-2 gene (Chromosome 19) used in these analyses are: probe 1 cg22282590 (position: 17514117), probe 2 cg07839313 (position: 17514600), probe 3 cg12090003 (position: 17516282), probe 4 cg16363586 (position: 17516329), probe 5 cg11558551 (position: 17516442), probe 6 cg01254505 (position: 17516470), probe 7 cg01329005 (position: 17516712), probe 8 cg09993699 (position: 17517008) and probe 9 cg20092122 (position: 17517221). Probe sequences can be downloaded at Illumina Infinium HumanMethylation450K Bead Chip product page at http://support.illumina.com/array/array_kits/infinium_humanmethylation450_beadchip_kit/downloads.html. Samples were divided into indicated categorical groups using the Biotab clinical information available at the TCGA DCC (https://tcga-data.nci.nih.gov/tcga/). Differences in sample number in figures are a result of sorting by categorical data, i.e. primary tumor samples that have PAM50 subtypes are less than the total number of primary samples with RNAseq expression. Expression values were also sorted by sample type, PAM50 subtype from RNAseq (TCGA AWG), and pathological stage. Analysis of BST-2 expression from different breast cancer subtypes was performed only with normal and primary tumor samples. Data from metastatic tumors were excluded from those analyses (<10 metastatic samples), but were used for the analysis of BST-2 expression between normal mammary tissue (Normal), primary tumors (Tumor), and metastatic tumors (Metastatic) (Fig 1D). All TCGA data was processed and analyzed using Graph Prism software. In addition, five datasets from the Gene Expression Omnibus (http://www.ncbi.nlm.nih.gov/geo/) were used: 1) GEO dataset GSE10797 [40] was used to analyze BST-2 and APOBEC3G (A3G) expression in the epithelium and stroma from normal and cancer tissues of patients undergoing surgical resection or reduction mammoplasty, 2) expression of BST-2 in different human breast cancer cell lines was analyzed using the dataset GSE41313 (only data for MCF-7, T47D, MDA-MB-231, and SUM-159 cells were downloaded) [41], 3) methylation and 4) expression pattern of the BST-2 gene in different human breast cancer cell lines was analyzed with GEO datasets GSE49794 and GSE45732, respectively. These datasets encompass a data superseries derived from the same cells and the same experiment [42], and 5) the effects of 5-aza-2’-deoxycytidine (DAC) treatment on BST-2, Claudin-6, and GAPDH expression in several human breast cancer cell lines was evaluated with the dataset GSE28976 (for HMLE, SK-BR-3 and MDA-MB-231 cells only) [43] and GSE36683 [44] (for MCF-7 cells only). Control and DAC treated samples were used. Cell line methylation versus expression analysis was conducted using the same methylation probe IDs listed previously. Methylation probes are reported as either independent beta-values or the average of probes 3–9 beta-values where indicated. Expression was derived from RNAseq data of GSE45732 and reported in reads per kilobases per million reads mapped (RPKM). All GEO data sets were processed and analyzed using Graph Prism software.

### 5-Azacytidine treatment and Flow Cytometry

Cell lines of interest were plated at 150,000 cells/well in a 6-well plate and treated with DMSO (vehicle) or 1 μM of 5-azacytidine (5-azaC, Sigma Aldrich) for 5 days. Cells were harvested using 0.25% trypsin-EDTA (Mediatech, Corning, NY, USA). Post-incubation, 8 ml of 10% FBS-containing RPMI media (Life Technologies) was added and cells were centrifuged. Media was aspirated and individual cells were resuspended in 2% FBS-containing PBS (Life Technologies). Cell suspension was passed through a 40 μM cell strainer (Falcon). Cells were incubated at 4°C for 1 hour with APC-conjugated anti-BST-2 primary antibodies or appropriate IgG (Ebioscience) and washed with 1x PBS. After washing, cells were incubated with the fluorescent intercalator—7-aminoactinomycin D (7-AAD) (BioLegend) at 4°C for 30 minutes to exclude dead cells. Stained cells were quantified using a FACS Calibur flow cytometer (BD). 10,000 events were collected per sample and FACS data were analyzed and plotted using Flowjo software (TreeStar).

### 5-Azacytidine treatment and reverse transcriptase quantitative real time PCR (RT-qPCR)

Human normal and breast cancer cell lines were plated at 150,000 cells/well in a 6-well plate and treated with DMSO (vehicle) or 1 μM of 5-azacytidine (5-azaC, Sigma Aldrich) for 5 days. Cells were lifted using Versene (a gentle cell dissociator, Life Technologies), washed with PBS, pelleted and stored at -20°C until required for analysis. RNA was isolated from frozen cells using the RNeasy mini kit (Qiagen) according to manufacturer's instructions. Equivalent amounts of RNA were treated with DNase (Qiagen). A portion of RNA was subjected to cDNA synthesis (ABI) as previously described [12,45–47]. RNA concentration and purity were assessed at 260/280 nm using the spectrophotometer. Using synthesized cDNA, sequence-specific primers were used to amplify BST-2 [4] and GAPDH [13,48]. Claudin-6 was amplified with (F: GGAGGAGAAGGATTCCAAGG, R: AGCCACCAGGGGGTTATAGA) primer pair. RT-qPCR was carried out with an ABI 7500 FAST thermal cycler in triplicates as previously described [11–13,45,46,48–50].

### Statistical analysis

Statistical analysis of significant differences was performed with Unpaired t test with Welch's correction (GraphPad Prism software). Error bars represent standard error of the mean (SEM) or 95% confidence interval (CI) of the mean. Correlation studies were carried out using GraphPad Prism software to calculate r^2^ and p values. r^2^ values of -0.30 or lower (inverse correlations) were considered significant. A p value of 0.05 or lower was considered significant.

## Results

### BST-2 is differentially expressed in various cancers

To understand the spectrum of BST-2 expression in various cancers, we analyzed the expression pattern of BST-2 mRNA in various tumors across the TCGA. Results reveal that levels of BST-2 expression in various cancer types differ. Compared to normal tissues, BST-2 expression in tumors is either unchanged (Fig 1A), significantly downregulated (Fig 1B), or significantly elevated (Fig 1C). In breast cancer, BST-2 expression is highest in metastatic tumors compared to primary tumors, and normal mammary gland tissues have the lowest expression (Fig 1D). Evaluation of BST-2 expression in different breast cancer subtypes show that while all cancer subtypes have elevated BST-2, tumors categorized as luminal B subtype has the highest BST-2 level compared to normal mammary tissue (Fig 1E). Although BST-2 expression in HER2 and basal tumor subtypes is not statistically different from normal tissues, it is well stablished that BST-2 is overexpressed in the most aggressive forms of breast cancer [4,30]. The lack of significance could be due to the high variability within the basal group (Fig 1E) and fewer data points for HER2 subtype. In addition, data points used in our analysis came from primary tumors only because metastatic samples within each subtype were excluded since there were insufficient data points for metastatic tumors.

Comparison of BST-2 expression levels in the different stages within the different subtypes show significant disease stage-dependent differences in BST-2 mRNA in the luminal tumor types compared to normal breast tissue (Fig 1F and 1I). However, BST-2 expression in the different disease stages of HER2 (Fig 1H) and basal (Fig 1I) tumor types was not different from normal tissues except for a modest significant difference (*p< 0*.*0313*) in stage II of basal tumors (Fig 1I). Host dependent variability in BST-2 levels and/or the small number of data points may have affected statistical analysis. Data presented in Fig 1E to 1I do not include metastatic tumors because there were few data points available.

To evaluate the level of BST-2 expression in different metastatic cells, we utilized breast cancer cell lines that originated from various metastatic sites. BST-2 expression both at the RNA (Fig 1J) and protein (Fig 1K) in different metastatic cells directly correlates to their aggressiveness. As such, triple-negative MDA-MB-231 cells presented the highest BST-2 levels followed by HER2 metastatic SK-BR-3 cells, while the luminal cell lines (MCF-7 and BT-474) express lower BST-2 levels (Fig 1J and 1K). These data support the premise that BST-2 levels are elevated in primary and metastatic breast tumor tissues and cell lines.

### *BST-2* is hypomethylated in breast tumors

**Fig 2:**
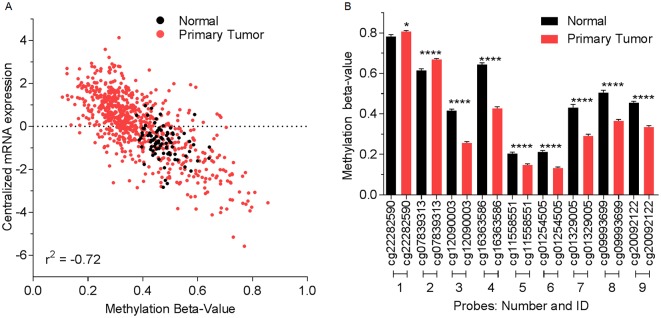
BST-2 DNA in breast tumors is hypomethylated compared to normal tissues. (A) BST-2 expression versus methylation plot among all primary tumor (red) and normal (black) breast tissues from BRCA in the TCGA. Beta-value ranges from 0 to 1. Beta-values closer to one depict hypermethylation and closer to 0 depict hypomethylation. (B) Methylation status among different CpG sites in primary tumor (red) and normal (black) breast tissues from invasive breast cancer bearing patients (TCGA). Significance was taken at P<0.05 (*) and P<0.001 (****). Error bars correspond to SEM.

To probe into the regulatory mechanism of BST-2 overexpression in breast tumors, we analyzed BST-2 methylation beta-values from paired tumor and normal breast tissues. The methylation beta-value was plotted against the corresponding RNA expression. Results reveal that BST-2 mRNA expression is inversely correlated to its DNA methylation status (Fig 2A) with a highly significant r^2^ value of -0.720. The inverse correlation of BST-2 mRNA with BST-2 DNA methylation beta-value signifies that in tumors, *BST-2* is hypo- or demethylated. This prompted us to query the differences in *BST-2* methylation across the entire gene. Comparing paired tumor and normal mammary tissues from the TCGA repository, we found that 7 CpG sites represented by probes 3 to 9 (see methods section for probe IDs) were significantly hypomethylated in tumors compared to normal tissues (Fig 2B). In contrast to probes 3 to 9, methylation beta-values for probes 1 and 2 show that these CpG sites are hypermethylated in breast tumors. Overall, these data show that BST-2 DNA is hypomethylated in mammary tumors compared to normal mammary tissue.

### Hypomethylation of specific CpG sites correlate with BST-2 expression in tumors

**Fig 3:**
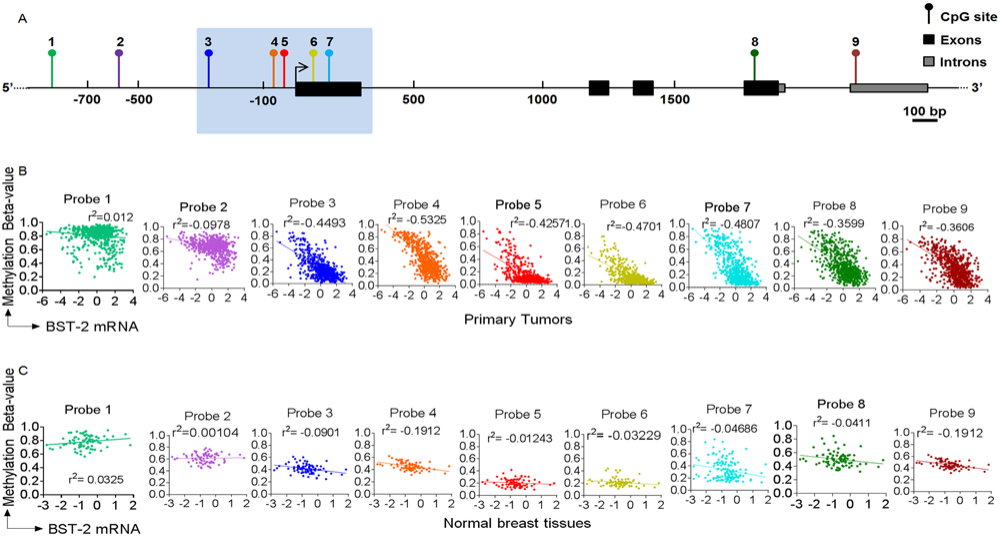
Methylation drives BST-2 expression in breast tumors, but not in normal breast tissue. (A) Location of the nine probes included in the Human Methylation 450 array that are associated with the BST-2 gene. Introns are solid gray rectangles while exons are solid black rectangles. (B) and (C) Correlation analysis between BST-2 mRNA levels and methylation values of probes 1 to 9 in (B) primary tumors and (C) normal breast tissues from TCGA. Best-fit line and r^2^ values are shown for each probe.

To better understand the effect of *BST-2* methylation on BST-2 expression, we sought to identify CpG sites in the BST-2 gene (Fig 3A), wherein methylation status was associated with elevated BST-2 expression in tumors compared to normal breast tissues. Fig 3A portrays the location of the different probes used in the Human Methylation 450 array across the BST-2 gene as described in the methods section. CpG sites corresponding to probes 3 to 7 which are proximal to the BST-2 gene transcription start site (TSS) had the lowest R-squared (r^2^, ranging from -0.4257 to -0.5325) values when plotted against corresponding BST-2 expression in primary tumors (Fig 3B). In addition, CpG sites downstream (probes 8 and 9) of the TSS did show a significant inverse correlation with tumor tissue BST-2 mRNA expression based on r^2^ values of -0.3599 to -0.3606 (Fig 3B). Moreover, CpG sites upstream (probes 1 and 2) of the TSS are hypermethylated (Fig 2A and 2B) and did not show a strong correlation with tumor tissue BST-2 mRNA expression based on r^2^ values of -0.0978 to 0.012 (Fig 3B). In parallel, correlation between BST-2 methylation beta-values and BST-2 mRNA in normal breast tissues was performed. There was no inverse correlation between CpG methylation and BST-2 expression with any of the 9 probes; r^2^ values range from -0.1912 to 0.03254 (Fig 3C). These data suggest that the CpG sites proximal to BST-2 gene TSS and downstream of the TSS may be responsible for transcriptional regulation of BST-2 in breast tumors.

### Hypomethylation of CpG sites proximal to the BST-2 promoter correlate with BST-2 overexpression in different breast tumor subtypes

**Fig 4:**
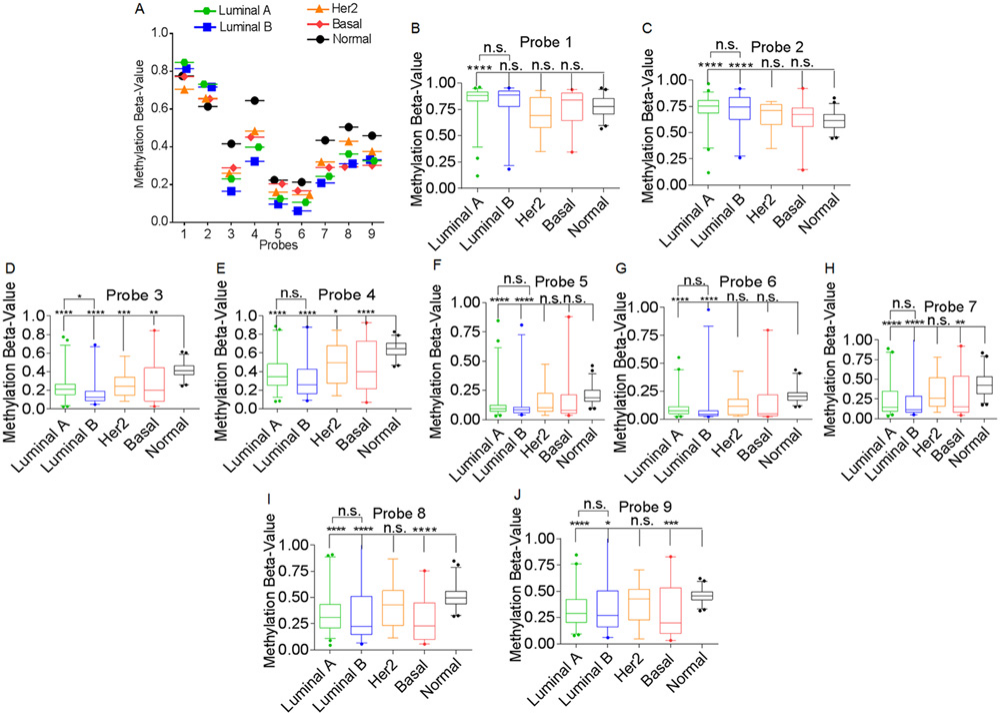
BST-2 DNA methylation pattern in different breast tumor subtypes. (A) Methylation status among different CpG sites from different breast cancer subtypes and normal breast tissue. Data does not include metastatic tumors. (B) to (J) Box plots of methylation values among different breast cancer subtypes and normal tissues corresponding to (B) probe 1, (C) probe 2, (D) probe 3, (E) probe 4, (F) probe 5, (G) probe 6, (H) probe 7, (I) probe 8, (J) probe 9. For statistical analysis, each subtype was compared to the normal breast tissue. The luminal subtypes were compared to each other. Significance was taken at P<0.05 (*), P<0.003 (**), P<0.0005 (***), and P<0.0001 (****). Error bars correspond to 95% CI.

Since demethylation of CpG sites strongly associates with increased expression of BST-2 in tumor tissues, we next analyzed the methylation level of BST-2 in different breast cancer subtypes. We found that *BST-2* is hypomethylated on the CpG sites represented by probes 3 to 9 irrespective of tumor subtype (Fig 4A). To an extent, BST-2 expression could be predicted by the degree of methylation of these probes (Fig 4A). As such, CpG sites represented by probes 1 and 2 do not mirror BST-2 expression in tumors and were hypermethylated (Fig 4A to 4C). For example, in luminal A tumors, CpG sites represented by probes 1 and 2 are hypermethylated compared to normal breast tissues. Luminal B tumors have the highest level of BST-2 mRNA (Fig 1C), and presents the lowest methylation values among probes 3, 4, 5, 6, and 7 (Fig 4A to 4H). Compared to normal breast tissues, the luminal tumor types (A and B) are differentially methylated across all probes compared to normal tissues (Fig 4B to 4J). Probes 3 to 9 are hypomethylated in luminal tumors compared to normal tissues while probes 1 and 2 in these tumors are hypermethylated compared to normal tissues, except for probe 1 in luminal B tumors that has a non-significant methylation beta-value compared to normal tissues (Fig 4B). The HER2 tumor type is hypomethylated at CpG sites represented by probes 3 and 4 (Fig 4D and 4E); while the basal tumor subtype is hypomethylated at CpG sites corresponding to probes 3, 4, 7, 8, and 9 (Fig 4D, 4E, 4H to 4J). These data suggest that *BST-2* methylation pattern at different CpG sites could predict breast cancer subtype (Table 1).

**Table 1:** Profile of BST-2 DNA hypomethylation in breast cancer subtypes as ranked by significant difference compared to normal breast tissue.

Probe ID	Probe name/ CpG site	Luminal A	Luminal B	HER2	Basal-like
**cg22282590**	**Probe 1**	**Hyper (** **** **)**	**unchanged**	**unchanged**	**unchanged**
**cg07839313**	**Probe 2**	**Hyper (** **** **)**	**Hyper (** **** **)**	**unchanged**	**unchanged**
**cg12090003**	**Probe 3**	**Hypo (** **** **)**	**Hypo (** **** **)**	**Hypo (** *** **)**	**Hypo (** ** **)**
**cg16363586**	**Probe 4**	**Hypo (** **** **)**	**Hypo (** **** **)**	**Hypo (** * **)**	**Hypo (** **** **)**
**cg11558551**	**Probe 5**	**Hypo (** **** **)**	**Hypo (** **** **)**	**unchanged**	**unchanged**
**cg01254505**	**Probe 6**	**Hypo (** **** **)**	**Hypo** (**** **)**	**unchanged**	**unchanged**
**cg01329005**	**Probe 7**	**Hypo (** **** **)**	**Hypo (** **** **)**	**unchanged**	**Hypo (** ** **)**
**cg09993699**	**Probe 8**	**Hypo (** **** **)**	**Hypo (** **** **)**	**unchanged**	**Hypo (** **** **)**
**cg20092122**	**Probe 9**	**Hypo (** **** **)**	**Hypo (** * **)**	**unchanged**	**Hypo (** *** **)**

* Is degree of hyper- or hypo-methylation based on statistical significance relative to normalbreast tissues (unpaired t test with Welch's correction). P value (*), where:

* is p < 0.02,

** is p < 0.003,

*** is p < 0.0005 and

**** is p < 0.0001.

### BST-2 mRNA expression correlates with DNA hypomethylation in breast cancer epithelial cells

**Fig 5:**
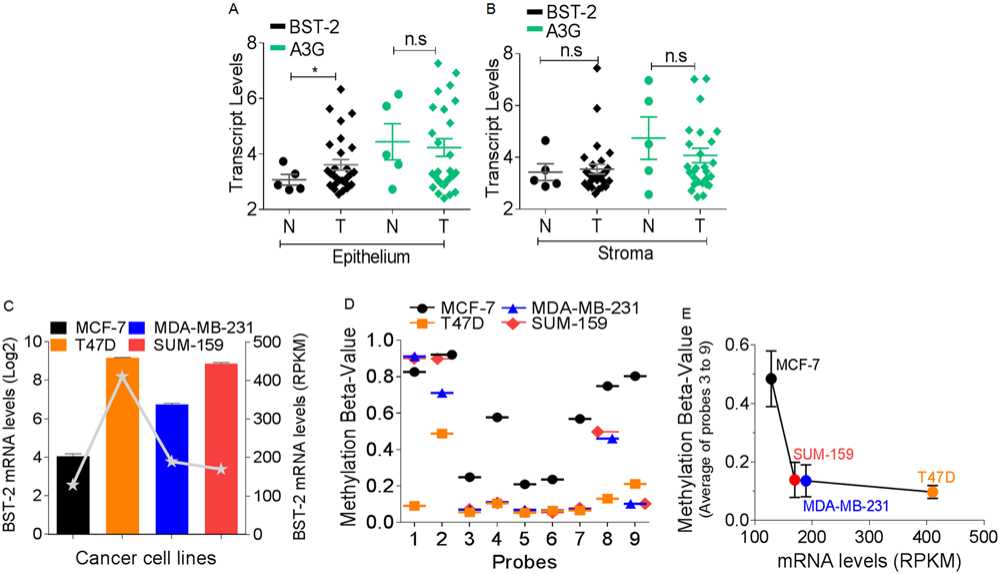
BST-2 expression inversely correlates with methylation density in neoplastic breast epithelial cell lines. (A) BST-2 and APOBEC3G (A3G) transcript levels in normal and tumor epithelial cells from patients with breast cancer. (B) BST-2 and A3G transcripts present in normal and tumor stromal cells of patients with breast cancer. Data were downloaded from GEO dataset GSE10797. (C) BST-2 expression levels among two luminal A cell lines (MCF-7 and T47D) and two triple-negative cell lines (MDA-MB-231 and SUM-159). Data were acquired from GEO dataset GSE41313 (bar graph, Log2 units) and GEO dataset GSE45732 (gray line graph, RPKM units). (D) Methylation status among different CpG sites from the breast cancer cell lines whose mRNA is presented in line graph in 5C. Data acquired from GEO dataset GSE49794. (E) BST-2 expression versus methylation plot among the four breast cancer cell lines presented in 5C (line graph) and 5D. Methylation beta-value is an average of probes 3 to 9 ± SEM. Methylation data was from GSE49794 (beta-value) and expression data was from GSE45732, a superseries from the same cells. Significance was taken at P<0.05 (*). n.s = not significant. Error bars correspond to standard error of the mean (SEM).

In breast carcinomas, neoplastic epithelial cells coexist and interact with various stromal cells that together create the tumor microenvironment. While neoplastic epithelial cells have higher BST-2 mRNA compared to normal cells, there was no difference in the expression pattern of another antiviral gene called Apobec3G (A3G) in these epithelial cells (Fig 5A). This data support the preposition that neoplastic epithelial cells are the source of elevated BST-2 expression in tumors [4]. Analysis of the stromal cells for BST-2 and A3G expression confirmed that cancer epithelial cells are the source of elevated BST-2 in tumors (Fig 5B). On the basis of this finding, we assessed correlation in patterns of BST-2 mRNA expression and *BST-2* methylation in well-established breast cancer cell lines downloaded from GEO dataset GSE10797 [31]. Our analysis revealed that elevated, levels of BST-2 mRNA varied among different breast cancer cell lines; with MCF-7 cells expressing the least BST-2 mRNA (Fig 5C, bar graph (GSE10797) and line graph (GSE49794)). Importantly, the relative levels of BST-2 expression are similar between the two datasets used (Fig 5C, bar and line graphs). As expected, level of BST-2 expression was inversely correlated to the methylation status of CpG sites represented by probes 3 to 9 (Fig 5C line graph, and 5D). As such, MCF-7 cells which have the lowest BST-2 mRNA levels (Fig 5C) have the highest methylation beta-values on probes 3 to 9 than any of the other cell lines (Fig 5D and 5E). Remarkably, high BST-2-expressing luminal A T47D cells, triple-negative MDA-MB-231 cells and basal SUM-159 cells are significantly hypomethylated (methylation beta-values of 0.21127, 0.10207 and 0.10295, respectively) on CpG sites corresponding to probe 9 (Fig 5D).

Moreover, the average methylation beta-value for probes 3 to 9 was inversely correlated to their corresponding BST-2 mRNA levels among all breast cancer cell lines analyzed (Fig 5E), providing additional support for a link between BST-2 expression and methylation status. Together with tumor data presented in Fig 4 and Table 1, it appears that BST-2 expression is increased by hypomethylation of CpG sites adjacent and downstream of the BST-2 TSS. Furthermore, in aggressive breast cancers and cancer cell lines, such as basal and triple-negative tumors, *BST-2* is hypomethylated at CpG sites downstream of the BST-2 TSS.

### Cancer cells with elevated BST-2 levels are unresponsive to 5-azacytidine induced BST-2 DNA demethylation

**Fig 6:**
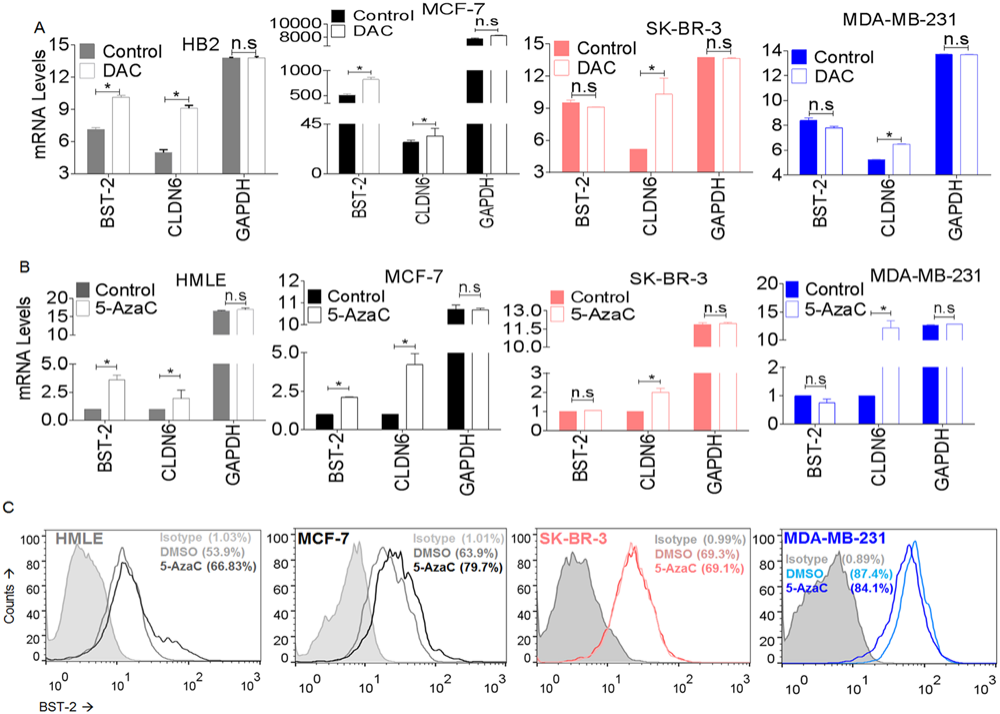
The effect of demethylating agents on BST-2 expression. (A) Meta-analysis of BST-2, Claudin-6 (CLDN6), and GAPDH expression levels in HB2, SK-BR-3, MDA-MB-231 cells (downloaded from GEO dataset GSE28976) and MCF-7 cells (downloaded from GEO dataset GSE36683) following treatment with 5-aza-2’-deoxycytidine (DAC). MCF-7 data was acquired from a different dataset, hence the difference in expression values. (B) BST-2, Claudin-6 (CLDN6), and GAPDH expression levels in HMLE, MCF-7, SK-BR-3, and MDA-MB-231 cells following treatment with DMSO (vehicle) or 1 uM of 5-azacytidine (5-AzaC) for 5 days. (C) BST-2 surface expression from HMLE, MCF-7, SK-BR-3 and MDA-MB-231 cells treated with DMSO (vehicle) or 1 uM of 5-azacytidine (5-AzaC) for 5 days as determined by flow cytometry. Numbers in parenthesis correspond to BST-2 expression presented as a percentage. Significance was taken at P<0.05 (*). Error bars correspond to SEM. n.s = not significant.

2′-deoxy-5-azacytidine (decitabine, DAC) is a deoxycytidine which incorporates into replicating DNA and prevents DNA methylation, thus, resulting in DNA hypomethylation and upregulation of gene expression. Since levels of BST-2 expression varies among cancer cells, we predicted that treatment with demethylating agents will further elevate BST-2 expression in cancer cells such as MCF-7 that express low BST-2, but that such treatment will have no effect on high BST-2 expressing cells. For this purpose, we used GEO datasets GSE28976 [43] and GSE36683 [44] (for MCF-7s only) to analyze the effect of DAC on *BST-2* methylation in several human breast cancer cell lines. We found that DAC treatment of normal breast cell line HB2 and low BST-2-expressing luminal A cell line MCF-7 led to increased BST-2 mRNA expression (Fig 6A). However, DAC-treated high BST-2-expressing HER2 SK-BR-3 and triple-negative MDA-MB-231 cells did not show any increase in BST-2 levels (Fig 6A). Importantly, mRNA levels of the tumor suppressor gene claudin-6 (CLDN6) reported to be increased in breast cancer cells upon DAC treatment [51] were induced in all cell types regardless of their subtype classification, while GAPDH mRNA, a house keeping gene did not change following DAC treatment (Fig 6A).

GEO data were validated by treating cells with the nucleoside analogue 5-azacytidine (azacytidine, 5-AZaC) and analyzing BST-2, CLDN6 and GAPDH mRNA levels (Fig 6B) and BST-2 protein levels (Fig 6C). In agreement with the GEO datasets in Fig 6A, HMLE (normal breast epithelial cell line) and MCF-7 cells treated with 5-AzaC showed a significant increase in BST-2 levels both at the RNA and protein levels (Fig 6B and 6C), while levels of BST-2 mRNA and protein following 5-AzaC treatment were unchanged in high BST-2-expressing HER2 SK-BR-3 and triple negative MDA-MB-231 cells (Fig 6B and 6C). As expected, CLDN6 was induced in all cell types upon 5-AzaC treatment (Fig 6B), but GAPDH did not change (Fig 6B). These results suggest that DAC/5-AzaC does not impact BST-2 expression in cancer cells with elevated BST-2 [52,53].

## Discussion

DNA demethylation was the first described epigenetic modification observed in various human cancers compared to normal tissues [54]. Cancer-linked DNA demethylation is associated with metastases of primary tumors [55,56] and is as prevalent as cancer-associated DNA hypermethylation. In cancer genomes, DNA hypermethylation is thought to occur in the promoter regions of tumor suppressor genes, which may lead to silencing of these tumor suppressors [57]. In contrast, DNA hypomethylation frequently occurs in DNA repeats, resulting in genomic instability and mutation in cancer genomes [58–60]. It has been suggested that hypomethylation of immunity-related genes, such as BST-2 may promote carcinogenesis. As such, promoter hypomethylation of IL-10 activates its expression and inhibits the generation of immune response against breast cancer [61], while hypomethylation of the immunogenic antigen SPAN-Xb may result in de novo B-cell response in myeloma cells [62]. In this study, we conducted meta-analysis of the methylation status of the BST-2 gene because BST-2 has been associated with development and progression of breast cancer in vivo [4]. The mechanism for the role of BST-2 in the evolution/progression of breast carcinogenesis is still poorly understood. Nevertheless, RNAi-mediated downregulation of BST-2 increases the survival of tumor-bearing mice [4], suggesting therapeutic significance.

Meta-analyses of human epidemiological data revealed that in breast tumors and neoplastic epithelial cells, BST-2 expression is epigenetically regulated by DNA demethylation. There are unique CpG sites corresponding to probes 3 to 9 from the Human Methylation 450 array and proximal to the BST-2 gene TSS that were demethylated across all breast tumor types (Fig 4A), irrespective of their subtype classification. In luminal B tumors, CpGs represented by probes 3 to 9 are significantly hypomethylated compared to normal tissues (Table 1); this observation is interesting given that this cancer subtype is considered to have a hypermethylated phenotype among breast cancer subtypes [63]. The CpG sites corresponding to probes 8 and 9 that are downstream and distal to BST-2 TSS were of significance to the triple-negative tumor subtype, as its methylation beta-value was lower compared to other subtypes (Figs 4A, 4I, 4J, 5D, and 5E). In contrast, the hypermethylated CpG sites represented by probes 1 and 2 that are upstream and distal to BST-2 TSS may have little or no effect on BST-2 regulation (Fig 4A to 4C).

Remarkably, the demethylated CpG sites represented by probes 3 to 7 are largely located adjacent to transcription factor binding sites in the BST-2 gene, including those of nuclear factor of activated T-cells (NF-AT), interferon regulatory factor (IRF) [12,64], signal transducers and activators of transcription (STAT), and nuclear factor kappa B (NF-кB) [65]. This observation is interesting because DNA methylation controls gene transcription through interference with the ability of transcription factors to bind to DNA [66]. A phenomenon that could partially explain the significant inverse relationship between BST-2 expression and BST-2 DNA methylation observed on probes 3 to 7. In addition, the inability of high BST-2 expressing MDA-MB-231 and SK-BR-3 cells to respond to DAC- or 5-AzaC-mediated induction of BST-2 expression suggests that high BST-2-expressing aggressive breast cancers at some point may have lost methylation-dependent regulation of BST-2 transcription which results in BST-2 overexpression and promotion of breast malignancy [4]. An alternative explanation could be that demethylating agents had a stabilizing effect on BST-2 in these cells, a phenomenon reported previously for MATN4 and CTSL2 unresponsiveness to 5-AzaC [52,53].

Examples of tumor-related overexpressed genes which become promoter hypomethylated during carcinogenesis includes, but not limited to, Sonic Hedgehog [67], P-cadherin [68], and CDH3 [69], as well as MATN4 and CTSL2 [52,53], supporting the data reported here for BST-2. Indeed, BST-2 overexpression due to DNA hypomethylation has been reported for glioblastoma [70] and lupus [71]. Patients with lupus presented with BST-2 hypomethylation on probes 1 to 7 compared to the controls, pointing to a common mechanism of methylation-dependent BST-2 regulation.

However, we cannot rule out other epigenetic-dependent or—independent sources of BST-2 regulation such as gene amplification, histone posttranslational modifications, increased translation of BST-2 or a decrease in the rate of BST-2 degradation or turnover. Further research is warranted to determine whether there are other mechanisms controlling BST-2 overexpression in breast cancer and whether methylation changes regulate BST-2 expression in other cancers including those in which BST-2 levels are unchanged (Fig 1A) or suppressed (Fig 1B) in tumor tissues compared to their corresponding normal tissues. Additionally, it is of interest to determine how changes in BST-2 DNA methylation pattern relate to the molecular pathology in breast cancer initiation, progression, and metastasis.

## Conclusions

We conclude that a greater frequency of *BST-2* hypomethylation was observed in breast cancer tissues and cells compared to normal breast tissues and cells. Therefore, BST-2 overexpression from DNA hypomethylation could influence breast carcinogenesis and could predict breast cancer prognosis or therapeutic response.
